# Identification of a chrXq27.3 microRNA cluster associated with early relapse in advanced stage ovarian cancer patients

**DOI:** 10.18632/oncotarget.401

**Published:** 2011-12-31

**Authors:** Marina Bagnoli, Loris De Cecco, Anna Granata, Roberta Nicoletti, Edoardo Marchesi, Paola Alberti, Barbara Valeri, Massimo Libra, Mattia Barbareschi, Francesco Raspagliesi, Delia Mezzanzanica, Silvana Canevari

**Affiliations:** ^1^ Depts. of Experimental Oncology and Molecular Medicine, Fondazione IRCCS Istituto Nazionale dei Tumori, Milan, Italy; ^2^ Department of Pathology and Oncologic Surgery, Fondazione IRCCS Istituto Nazionale dei Tumori, Milan, Italy; ^3^ Department of General Pathology, University of Catania, Italy; ^4^ Department of Pathology, Ospedale Santa Chiara, Trento, Italy; ^5^ Department of Oncologic Surgery, Fondazione IRCCS Istituto Nazionale dei Tumori, Milan, Italy

**Keywords:** advanced-stage ovarian cancer, early relapse, microRNA profiling, miR-506, cisplatin, miR-513a-5p, miR-513b

## Abstract

A major challenge in advanced-stage epithelial ovarian cancer (EOC) is prediction of chemoresistant relapse. Our aim was to identify a microRNA (miRNA) signature associated with early relapse in advanced-stage EOC patients. miRNA expression was assessed by microarray profiling in training (*n = 55*) and test (*n = 30*) sets selected on the basis of time to relapse (TTR), followed by internal quantitative reverse transcriptase-PCR validation on a set of 45 consecutive cases unselected for clinical response and external in silico validation on publicly available datasets. Thirty-two differentially expressed miRNAs in early vs. late relapsing patients were identified in the training set. In the test set, 8 of these, belonging to a cluster located on chrXq27.3, were down-modulated in early relapsing patients. Hierarchical clustering of the internal validation set according to chrXq27.3 miRNA expression associated low miRNA expression with shorter TTR (log-rank *P=0.00074*, HR 2.44). The cluster was an independent prognostic factor in both internal and external validation sets. Forced expression of chrXq27.3-cluster selected miRNAs in human EOC cellular models was associated to reduction of cell proliferation and increased sensitivity to cisplatin. The role of down-modulation of the chrXq27.3 miRNA cluster in early relapse of advanced-stage EOC patients and its association to a reduced sensitivity to chemotherapeutic treatments warrant further investigation.

## INTRODUCTION

Epithelial ovarian cancer (EOC) remains one of the most challenging areas of cancer research. Despite ongoing efforts to develop an effective screening strategy [1] and years of research into new treatments, the survival rate from EOC remains one of the lowest of all cancers [2]. The poor ratio of survival to incidence in EOC is a result of the high percentage of cases presenting at an advanced-stage; the overall 5-year survival rate is approximately 30% [1]. Standard treatment for advanced-stage EOC is aggressive surgery followed by platinum-taxane chemotherapy, with response rates of over 80% [3]. However, most of these patients will eventually relapse, with a median time to relapse (TTR) of 18 months. Several drugs are available to treat relapsing patients, although clinical responses remain short-lived and lead to only marginal improvements in survival of patients with platinum-resistant disease [4]. Therefore, there is a critical need for early identification of patients with drug-resistant cancers so that alternative therapeutic modalities can be offered.

Normal human cells express thousands of non-coding RNAs, including microRNAs (miRNAs), whose regulatory activity and alterations in expression contribute to the pathogenesis and progression of several human malignancies [5-7]. As recently reviewed [8], a general down-modulation of miRNA expression is observed in EOC compared to normal tissue, and the most frequently deregulated miRNAs are members of the let-7 and miR-200 families; the latter is involved in the epithelial mesenchymal transition and has been found to be de-regulated in both early- and advanced-stage EOC [9, 10]. Nonetheless, clear consensus miRNA signatures associated with EOC prognosis or prediction of response to conventional therapy have not yet described [8].

Recently, the results of an integrated genomic analyses of a large cohort of advanced-stage, high-grade serous ovarian cancer has become publicly available [11]; in this Cancer Genome Atlas study, clustering of miRNA expression data identified three subtypes of tumors but no data of association with early relapse are at present available.

With the objective of identifying a miRNA signature of EOC early relapse, a training set of samples from advanced-stage EOC patients, selected for different residual disease and TTR, and profiled by miRNA microarray analysis, has been characterized by a miRNA signature comprising a miRNA cluster located on Chromosome X and down modulated in early relapsing patients. A test set with similar clinical characteristics challenged with the identified miRNA signature confirmed that low expression levels of chrXq27.3 miRNAs cluster associated with an early relapse. Validation by quantitative reverse transcriptase-PCR (qRT-PCR) showed that expression of the chrXq27.3 miRNA cluster correlated with clinical outcome in a cohort of consecutively collected samples. The association of low expression of chrXq27.3 miRNA cluster with a shorter TTR was confirmed by in silico analysis of publicly available datasets of advanced stage EOC patients with known clinical history. Finally, the functional role of three miRNAs was investigated using two human EOC cell lines, in which reduced cell proliferation and increased sensitivity to cisplatin (DDP) treatment was demonstrated after forced miRNAs expression.

## RESULTS

### Identification of a miRNA profile associated with early relapse in advanced-stage EOC

**Figure 1:**
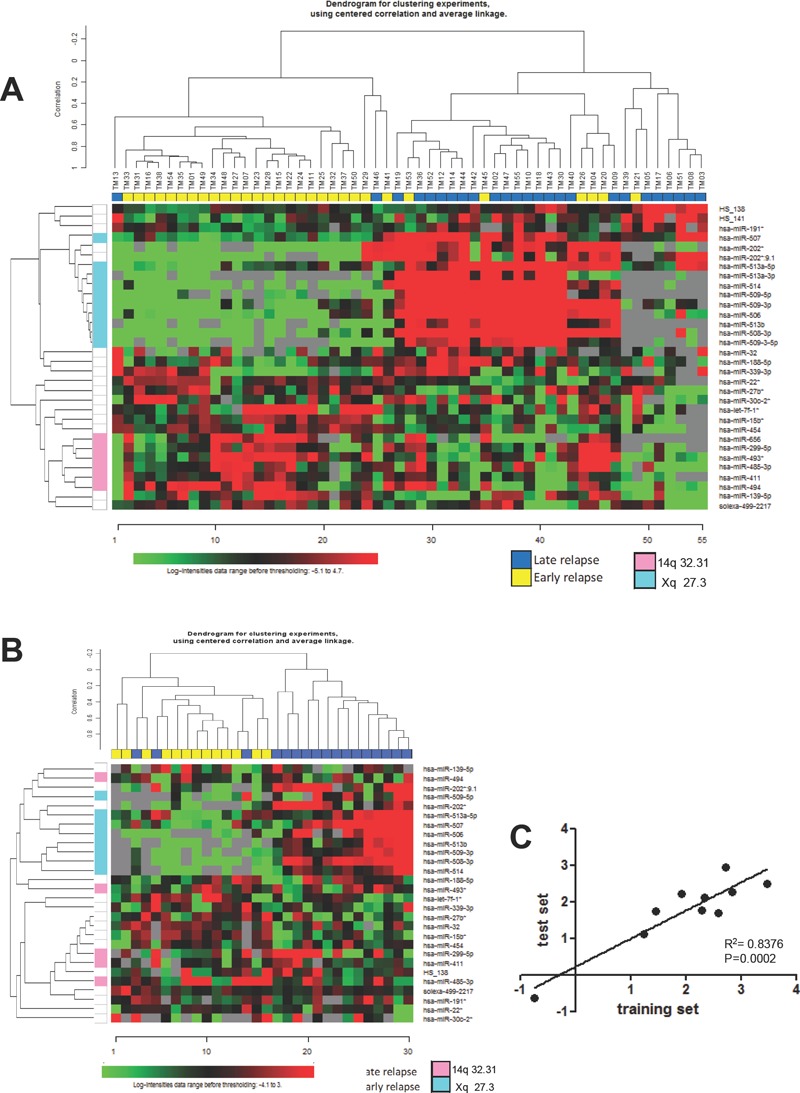
A miRNA expression profile distinguishes between EOC patients with early and late relapse A) Heat map of hierarchical clustering analysis of patients from the training set based on the 32 miRNAs differentially expressed at FDR<10%. B) Hierarchical clustering of patients from test set challenged with the miRNA profile identified in the training set. Twenty-eight of 32 miRNA were detected in the test set. A and B: columns, samples; rows, miRNA expression levels. Red, over-expressed miRNAs; green, under-expressed miRNAs. C) Correlation between training and test sets for the fold changes of the 10 miRNAs identified as differentially expressed in both sets and that maintained a P value <0.01 in the test set (see also Table 2).

The strategy for identifying a miRNA profile associated with early relapse was initially based on the selection of a training set of 55 advanced-stage EOC from patients with a different clinical history of response to first-line chemotherapy. Thirty patients had early relapse, and 25 patients had late relapse (Table 1). In this dataset, 744 miRNAs were detected and class comparison analysis, imposing a false discovery rate (FDR) <10%, identified an expression signature comprising 18 down-modulated and 14 up-modulated miRNAs in patients with early relapse (Figure 1A). Ten of the 18 down-modulated miRNAs were located on chromosome X (chrXq27.3, X: 146.27-146.36), and six of the 14 up-regulated miRNAs were located on chr14q32.31 (Table 2). Analysis of association between miRNA expression and clinical parameters (stage, grading and debulking) showed that, by class comparison and imposing a FDR<10%, no significant modulation of miRNA was detected between stage III and IV lesions, well and moderately versus poorly differentiated and undifferentiated tumors, or optimally debulked (OD) versus sub-OD patients.

**Table 1:** Clinical and Pathologic Characteristics

	Training set (n = 55)		Test set (n = 30)		Validation set (n = 45)	
	N°	%	N°	%	N°	%
**Age, years**						
median	56		53		50	
range	30-78		25-74		25-85	
**Histology**						
Serous	40	73	22	73	27	60
Undifferentiated	4	7	3	10	4	9
Clear Cells	3	5	_	_	3	7
Endometroid	3	5	5	17	4	9
Others + Mixed	5^a^	9	_	_	6^b^	13
NA	_	_	_	_	1	2
**Stage (FIGO)**						
III	45	82	25	83	37	82
IV	10	18	5	17	8	18
**Grade**						
1-2 well/moderately differentiated	7^c^	13	9^c^	30	7	15
3, poorly differentiated	43	78	18	60	33	74
Undifferentiated	4	7	3	10	4	9
NA	1	2	_		1	2
**Amount of residual disease**						
NED	12	22	3	10	6	13
<1 cm, mRD	12	22	8	27	16	35
≥1 cm, GRD	31	56	19	63	23	51
**Relapsing patients^d^**						
Early	30	54	13	40	not a priori selected	
Late	25	46	17	60	not a priori selected	

Abbreviations: NA, not available. FIGO, International Federation of Gynecological and Obstetrics staging system. NED: not evident disease; mRD: minimal residual disease; GRD: gross residual disease. Patients NED or with mRD are considered optimally debulked (OD).

a Including 4 mixed-type tumors and 1 Mullerian tumor.

b Including 5 mixed-type tumors and 1 mucinous tumor.

c Only 1 well-differentiated G1 case included.

d Early relapse: whitin 12 or 6 months from the end of therapy for OD and sub-OD patients, respectively. Late relapse: equal ol longer than 36 or 12 months from the end of therapy for OD and sub-OD patients, respectively.

**Table 2:** miRNAs differentially expressed between late and early relapsing patients in training and test sets

		Training set ^a^			Test set ^b^		
miRBase annotation	Illumina ID	Fold-change late/early relapse	Parametric p-value		Fold-change late/early relapse	Parametric p-value	Map Location
**hsa-miR-513b**	**ILMN_3168868**	**11.14**	**1.90E-06**		**5.67**	**0.00017**	**chrXq27.3**
**hsa-miR-514**	**ILMN_3168464**	**7.17**	**2.60E-06**		**4.84**	**3.00E-07**	**chrXq27.3**
**hsa-miR-508-3p**	**ILMN_3168488**	**6.63**	**3.17E-05**		**7.71**	**5.00E-07**	**chrXq27.3**
hsa-miR-202*:9.1	**ILMN_3167129**	**6.04**	**2.65E-05**		**3.2**	**0.00012**	**NA**
hsa-miR-509-3-5p	ILMN_3168790	5.71	0.000392				chrXq27.3
hsa-miR-513a-3p	ILMN_3168779	5.23	0.000256				chrXq27.3
**hsa-miR-506**	**ILMN_3168328**	**5.07**	**0.0001**		**4.36**	**0.00318**	**chrXq27.3**
**hsa-miR-509-5p**	**ILMN_3168789**	**4.92**	**6.11E-05**		**3.41**	**0.00295**	**chrXq27.3**
hsa-miR-202*	ILMN_3168871	4.38	0.002787		2.33	0.052874	chr10q26.3
**hsa-miR-509-3p**	**ILMN_3168363**	**3.79**	**0.00093**		**4.65**	**0.0006**	**chrXq27.3**
**hsa-miR-507**	**ILMN_3167727**	**2.75**	**0.00011**		**3.31**	**2.17E-05**	**chrXq27.3**
**hsa-miR-513a-5p**	**ILMN_3168869**	**2.37**	**0.00061**		**2.15**	**0.00283**	**chrXq27.3**
HS_141	ILMN_3167414	1.92	7.50E-06				NA
hsa-miR-339-3p	ILMN_3168833	1.82	0.004224		1.01	0.969504	chr7p22.3
hsa-miR-32	ILMN_3167472	1.69	0.004243		0.79	0.113184	chr9q31.3
hsa-miR-191*	ILMN_3167124	1.5	0.001467		1.13	0.36892	chr3p21.31
HS_138	ILMN_3167622	1.45	0.001171		1.07	0.751857	NA
hsa-miR-188-5p	ILMN_3167745	1.44	0.003238		0.89	0.543601	chrXp11.23
hsa-miR-22*	ILMN_3168621	0.72	0.002515		0.97	0.850966	chr17p13.3
hsa-miR-27b*	ILMN_3168599	0.66	0.002082		0.71	0.077505	chr9q22.32
hsa-let-7f-1*	ILMN_3167319	0.6	0.002563		0.95	0.818997	chr9q22.32
hsa-miR-15b*	ILMN_3168693	0.59	0.00035		0.64	0.0061	chr3q25.33
hsa-miR-454	ILMN_3168319	0.57	0.001298		1.02	0.893015	chr17q.22
hsa-miR-411	ILMN_3167988	0.55	1.23E-05		1.36	0.058	chr14q32.31
hsa-miR-30c-2*	ILMN_3168727	0.55	0.002809		0.97	0.927898	chr6q13
hsa-miR-139-5p	ILMN_3166992	0.53	0.003255		0.87	0.678405	chr11q13.4
hsa-miR-299-5p	ILMN_3167913	0.51	0.000767		1.52	0.141925	chr14q32.31
hsa-miR-493*	ILMN_3167972	0.5	0.000377		1.12	0.472174	chr14q32.2
solexa-499-2217	ILMN_3168900	0.5	0.003835		0.91	0.289138	NA
hsa-miR-485-3p	ILMN_3168166	0.47	0.000859		0.74	0.303974	chr14q32.31
hsa-miR-656	ILMN_3168467	0.4	2.43E-05				chr14q32.31
hsa-miR-494	ILMN_3168446	0.35	1.13E-05		0.99	0.981626	chr14q32.31

a 32 miRNAs were differentially expressed at a false discovery rate < 10% and detection p-value < 0.05 between late or early relapsing patients: 29 were mature miRNAs from miRbase v12.0 and 3 were putative miRNAs derived from deep sequencing approaches (see description of Illumina platform on GEO repository; GEO accession: GPL8179).

b Empty cells: miRNAs identified in training set and filtered out in test set (detection p-values greater than the threshold adopted in the analysis for more than 50% of samples).In bold: differential expressed miRNAs validated in the test set.

A test set (13 and 17 patients with early and late relapse, respectively), selected in agreement with debulking status and TTR criteria adopted for the training set, was used to validate the microarray data obtained with the training set. As listed in Table 1, there were no differences in age, stage, grade, histology, or debulking status of patients between the training and test sets, and only the median follow-up period was longer in the latter (49 versus 64 months, respectively). Profiling of the test set detected 741 miRNAs, including 28/32 miRNAs found differentially expressed in the training set. Unsupervised hierarchical clustering analysis, based on the miRNA signature identified in the training set, correctly classified test set patients according to relapse in 90% of cases (27/30) (Figure 1B). Ten of these miRNAs showed significant differential expression (*P* <0.01), and all but one were down-regulated in patients with early relapse (Table 2). Among these, eight were located in chrXq27.3, representing a highly correlated and co-expressed miRNA cluster. The fold change of the 10 differentially expressed miRNAs between patients with early and late relapse was highly correlated (R^2^= 0.838) in the two clinical sets (Figure 1C).

**Figure 2:**
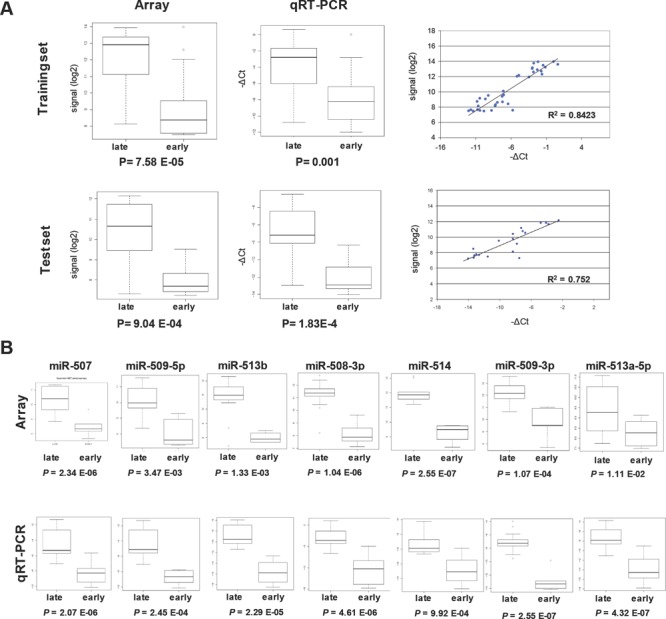
qRT-PCR validation of the chrXq27.3 miRNA cluster A) Comparison of miR-506 expression obtained by miRNA expression profile and qRT-PCR on 39 samples (17 early and 22 late relapse) from training set (upper panels) and 24 samples (10 early and 14 late relapse) from test set (lower panels). B) Comparison of chrXq27.3 miRNA cluster expression obtained by miRNA expression profile (upper panels) and qRT-PCR (lower panels) on the 24 samples from test set. *P* values of differential expression between late and early relapsing patients are reported.

Thirty-nine samples belonging to training set and 24 samples belonging to test set were analyzed by qRT-PCR for expression of the mature form of miR-506 showing an intermediate fold-change expression in training and test set. Microarray data were validated and a high correlation between data obtained with the two assays was observed in both training and test set (R^2^ was 0.8423 and 0.752 respectively; Figure 2A). Samples from test set were then analyzed by qRT-PCR for expression of the mature forms of the other miRNAs belonging to the chrXq27.3 cluster and down-regulated in early relapsing patients. As in the case of miR-506, the microarray and qRT-PCR showed a significant correlation median (R^2^ = 0.661; Figure 2A-B). miR-335*, not belonging to the chrXq27.3 cluster and not differentially expressed among the 744 miRNA detected in the training set, was selected and validated as unrelated control (R^2^ =0.498; data not shown).

### Down-regulation of chrXq27.3 cluster is associated with shorter TTR

**Figure 3:**
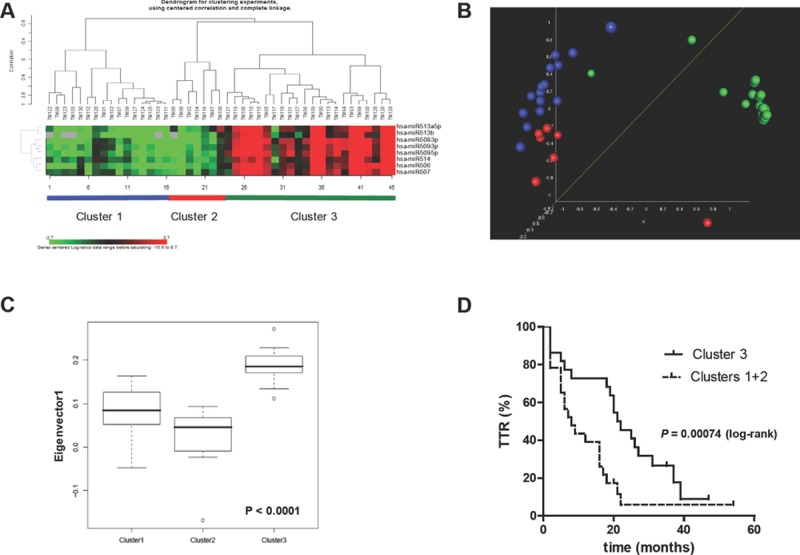
Down-regulation of chrXq27.3 miRNAs associated with shorter TTR A) Unsupervised clustering of validation set samples, according to chrXq27.3 miRNA expression by qRT-PCR. B) Multidimensional Scaling (MDS) analysis. MDS analysis preserves the pair-wise similarities between samples in a three-dimensional graphical representation without forcing the samples into specific clusters as done by hierarchical clustering. The *P*-value of global clustering test based on three components (covering 92% of total variation) obtained by Euclidean distance is <0.001. MDS was generated using uncentered correlation. C) Principal component analysis. The box plot shows the eigenvalues for the 3 clusters based on the first principal component accounting for 44.1% of the overall variability of the miRNA data across the 45 samples. Kruskal-Wallis test: *P*-value< 0.0001. D) Kaplan-Meier survival curves of patients included in the validation set stratified according to chrXq27.3 cluster classification, clusters 1 and 2 (dotted line) and cluster 3 (continuous line). Curves were compared using the log-rank test; *P* = 0.00074.

We then used qRT-PCR to analyze the expression of the 8 chrXq27.3 miRNAs in a third cohort of 45 advanced-stage consecutive EOC cases (validation set) that were not previously selected for response to first-line treatment (see Table 1). In this clinical set (median of follow-up period = 35 months), there were no differences in age, stage, grade, histology, or debulking status compared to the other cohorts. Unsupervised clustering classified validation set patients into three clusters (Figure 3A): clusters 1 and 2 (*n* = 16 and 7, respectively) both showed low expression of chrXq27.3 miRNAs, while cluster 3 (*n* = 22) had high expression of chrXq27.3 miRNAs. Clusters 1 and 2, as determined by both multi dimensional scaling (MDS) and principal component analysis (PCA) analyses, had a global expression comparable and distinct from cluster 3 (Figures 3B and 3C); thus, they were considered together in further analyses. Kaplan-Meier analysis indicated that patients belonging to clusters 1 and 2 experienced a shorter TTR (log-rank, *P* = 0.0007; HR = 2.44, 95%CI: 1.25-4.76). The median TTR was 8 and 21 months for patients belonging to clusters 1 and 2 (low chrXq27.3 miRNA expression) and cluster 3 (high chrXq27.3 miRNA expression), respectively (Figure 3D).

Using miRNA cluster expression and surgical debulking as covariates, a bivariable Cox regression analysis performed on the type II EOC subgroup of patients (*n* = 40, excluding samples with grade 1 tumors and clear cell or mucinous histotypes) indicated down-regulation of chrXq27.3 miRNAs as a possible independent prognostic indicator of early relapse (HR = 2.33; 95% CI: 1.06-5.12, *P* = 0.035). As expected, the prognostic relevance of surgical debulking was confirmed (HR = 4.3, 95% CI: 2.03-9.27, *P* = 0.00015) in this model.

### *In silico* validation of the prognostic impact of chrXq27.3 miRNAs

**Figure 4:**
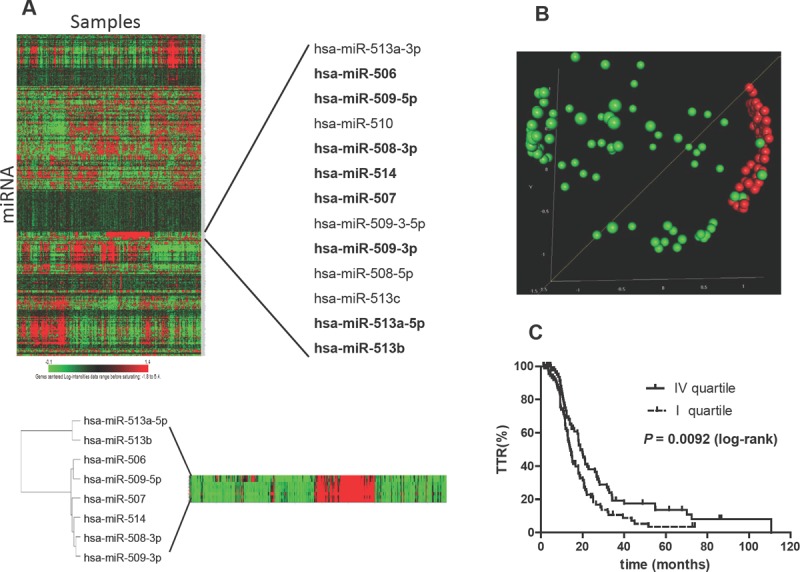
*In silico* analysis on TGCA dataset A) Unsupervised clustering of whole miRNome profile in the TGCA study. Only stage III and IV ovarian cancers with TTR data were included in the analysis and clustered in a heat map diagram. The heat map reveals the major miRNA patterns present in the array matrix and points out a highly correlated cluster of thirteen miRNAs all belonging to chrXq27.3. A magnification of the 8 chrXq27.3 miRNAs associated to TTR in our work is shown (samples were sorted following the order established in the unsupervised cluster). B) Multidimensional Scaling (MDS) analysis. Patients included in the TGCA dataset were stratified according to the first principal component (PC1) retaining most of the variability of the data (74%). Two groups of patients were included in the MDS analysis: first PC1 quartile (green) and last PC1 quartile (red). The MDS analysis was performed based on the expression of the 8 chrXq27.3 miRNAs. The P-value of global clustering test based on three components (covering 90% of total variation) obtained by Euclidean distance is <0.001. C) Kaplan-Meier survival curves related to the two sub-groups of patients. First PC1 quartile (dotted line) and last PC1 quartile (continuous line); curves were compared using the log-rank test; P = 0.0092.

The TGCA data set of miRNA profile [11] was used for external validation restricting the analyses to the 360 stage III and IV EOC samples for whom complete survival data are available. On this subset of samples the expression of all the 8 miRNAs belonging to chrXq27.3 was detected. Unsupervised analysis on the miRNome profile provided evidence that the miRNAs located on chrXq27.3 are members of a highly correlated and co-expressed miRNA cluster. In particular six out of eight chrXq27.3 miRNAs (miR-506, miR-507, miR-508-3p, miR-509-3p, miR-509-5p and miR-514) showed Pearson’s correlation greater than 0.95 (Figure 4A). Principal component analysis was applied on the expression of the 8 chrXq27.3 miRNAs and the first component (PC1) covering 74% of total variation in the data was used for survival analysis. Based on PC1, patients were split in quartiles and we considered two groups with low and high expression intensities of chrXq27.3 miRNA cluster corresponding to the first (n=90) and the fourth (n=90) quartile, respectively. The two groups represent patients with well-defined expression pattern as highlighted by MDS analysis (Figure 4B). Kaplan-Meier analysis confirmed that patients with low expression of chrXq27.3 miRNA cluster experienced shorter progression free survival (log-rank, P=0.0092; HR= 1.57, 95%CI: 1.12-2.22). The median TTP was 14 and 19 months for patients with low and high chrXq27.3 miRNA expression, respectively (Figure 4C). Using miRNA cluster expression and surgical debulking as covariates, a bivariable Cox regression analysis indicated down-regulation of chrXq27.3 miRNAs as a possible independent prognostic indicator of early relapse (HR = 1.85; 95% CI: 1.28-2.66, *P* = 0.001).

### Over-expression of cluster chrXq27.3 miRNAs in EOC cell lines: effects on survival and platinum sensitivity

The association of chrXq27.3 miRNA cluster lower expression with shorter TTR, suggested a possible involvement of these miRNAs in cell growth and response to therapy.

**Figure 5:**
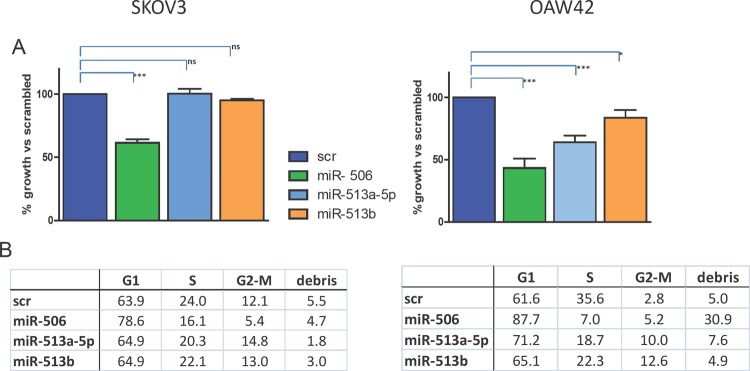
Effects of forced expression of chrXq27.3-cluster selected miRNAs on EOC cell line survival and cell cycle A) Percentage of cell viability evaluated by trypan blue exclusion assay 72 h after miR-506, miR-513a-5p or miR-513b transfection on SKOV3 and OAW42 cells compared to control-transfected cells (scr). Data are the mean ± SD of three experiments. *** : P < 0.005; ** P < 0.01. B) Effects on cell cycle dynamics on the same cells as assessed by cytofluorimetric analysis. A representative experiment of three performed is shown.

Three miRNAs belonging to the chrXq27.3 cluster, miR-513b, miR-506 and miR-513a-5p, were selected according to their different fold change expression observed by microarray and class comparison analysis on EOC samples (see Table 2) and their expression was forced by transient transfection in two EOC cell lines, SKOV3 and OAW42. The miRNAs increased expression level upon transfection and its specificity was demonstrated by qRT-PCR (supplementary Figure 1). Forced expression of miR-506 induced a significant reduction of cell proliferation in both cell lines (39±3% and 57±7%, relative to control cells transfected with the scrambled miRNA, in SKOV3 and OAW42 respectively), whereas miR-513a-5p and miR-513b showed a less intense, albeit significant anti proliferative effect (37±5% and 17±6% of reduction respectively) on OAW42 cells only (Figure 5A).

A relevant increase of cells blocked in the G1 phase in both cell lines (increase range 16% - 30% compare to control in three independent experiments) was observed upon forced expression of miR-506, while increase of cell death, evaluated as cellular debris, was observed in OAW42 cells only (Figure 5B). Forced expression of the other two miRNAs in SKOV3 cells, mirroring the marginal effects on cell proliferation, did not substantially affect cell cycle dynamics. In miR-513a-5p-transfected OAW42 cells, accumulation of cells in the G1-phase was evident and was accompanied with a slight increase (8%) of cells blocked in the G2-M phase. In agreement with the weaker anti-proliferative effect obtained on OAW42 cells after miR-513b-transfection, only a marginal increase (5%) of G1-phase and a 10% increase of cells blocked in G2-M phase was observed as compared to the other two miRNAs (Figure 5B).

**Figure 6:**
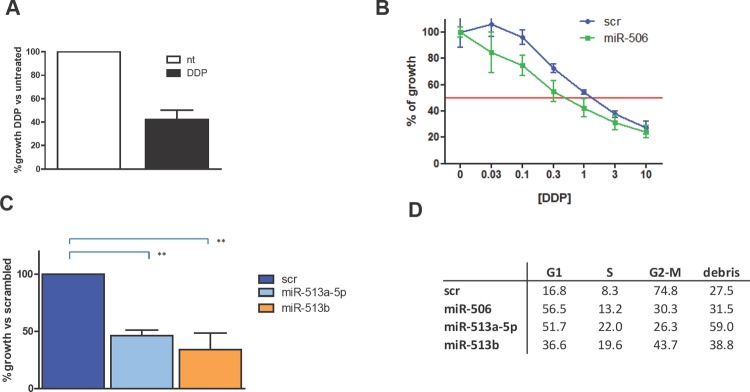
Forced expression of chrXq27.3-cluster selected miRNAs increased platinum sensitivity in OAW42 cells A. Percentage of cell viability of control-transfected cells (scr) OAW42 cells evaluated by trypan blue exclusion assay. Treated cells (black bar) were exposed to a DDP dose (1x10^−6^ M) corresponding to the IC_50_ of parental OAW42 cells. Open column represents the untreated (nt) control. Data are the mean ± SD of three independent experiments. B. Evaluation of DDP sensitivity tested in serial dilution from 10x10^−6^ to 0.03 x10^−6^ M in OAW42 cells 72 h after miR-506 transfection (miR-506) compared to control scrambled transfected cells (scr) as assessed by SRB assay. C. Percentage of cell viability evaluated by trypan blue exclusion assay 72h after miR-513a-5p or miR-513b transfection, compared to control-transfected cells (scr). Cells were exposed to 1 x10^−6^ M DDP. ** P < 0.01. D) Effects on cell cycle dynamics on the same cells as assessed by cytofluorimetric analysis. A representative experiment of three performed is shown.

Based on these data, OAW42 cell line was selected to assess the effect of the forced expression of the three miRNAs on response to DDP treatment. A growth inhibition of 58±8% (Figure 6A) was observed in scrambled miR-transfected (control) cells exposed to a DDP concentration that, according to our previous knowledge, corresponds to IC_50_ in parental cells. DDP sensitivity at concentration ranging from 10x10^−6^ to 0.03x10^−6^ M was significantly increased (P=0.001) as assessed by sulphorodamine-B (SRB) assay following forced expression of miR-506 (Figure 6B). Also miR-513a-5p and miR-513b caused a significantly increased DDP sensitivity. At a DDP concentration corresponding to the IC_50_ in scramble-transfected cells, cell growth was further inhibited following 54±5% following miR-513a-5p and 66±14% by miR-513b transfection (Figure 6C).

In miRNA-transfected-DDP-treated cells, the effects on cell cycle resulted more variable and less informative. As a general observation, all the three miRNAs induced, although at different extent, an accumulation of cells in G1-phase (figure 6D). Forced expression of miR-513b caused a higher accumulation of cells in G2-M phase as compared to miR-506 and miR-513a-5p whereas forced expression of miR513a induced a more consistent increase of cell debris as compared to the other two miRNAs.

## DISCUSSION

Therapeutic decisions in EOC after first-line chemotherapy should be based on the risk of tumor relapse. Although for solid tumors such as breast and prostate cancer, molecular profiling of primary tumors can help in defining risk of relapse, prediction of drug response, and time to relapse [12, 13], the heterogeneous clinical and pathological characteristics of advanced-stage EOC patients make it difficult to exploit genome-wide information to stratify patients according to prognosis [14]. However, there is accumulating evidence that miRNAs may be of higher utility than mRNA expression due to their master regulatory role, and examples of their utility as both biomarkers and therapeutic targets have been reported for other solid tumors [15]. To our knowledge, this is the first study to date specifically designed to identify miRNAs as markers of early relapse in advanced-stage EOC. Using 2 independent cohorts and microarray analysis we identified a cluster of miRNAs, located at chrXq27.3 in a region <0.5 Mb, that are always down-modulated or almost switched-off in patients that experience early relapse. Validation of this result by qRT-PCR in a set of consecutively collected cases highlighted a significant correlation between low expression of chrXq27.3 miRNAs and clinical outcome.

Our cohorts of patients, which were well-characterized for clinical and pathological characteristics, came from a single institution; therefore, efforts were focused in reducing the potential over-fitting of data [16]. Training and test sets, selected with the same criteria and analyzed on the same miRNA microarray, were independently obtained from institutional tissue bank and archival pathologic repository, respectively. Due to the fact that the quality of RNA extracted from FFPE tissues (test set) was not as high as that of RNA extracted from frozen tissues (training set), the number of samples in the test set was quite limited; however, both sets were equally reliable when used for qRT-PCR measurements. For validation of chrXq27.3 miRNA cluster, we used a third cohort of consecutive frozen samples from advanced-stage EOC and qRT-PCR as detection assay.

Our criteria for case selection only partially overlap those adopted in the Cancer Genome Atlas (TCGA) project for EOC which considered only clinically annotated stage II-IV high-grade serous ovary cancers [11]. In our analysis, the selection was driven by different TTR in stage III-IV cases without prior selection by grade and histotype. However, taking into account the significant progress in the histopathological sub-classification of EOC [17], the majority of our cases could be classified as so-called type II tumors (grade 2-3; subtype: serous, undifferentiated and endometrioid), characterized by very aggressive behavior [18]. Indeed, only 1/130 cases was grade 1, 5/130 were clear cells and 1/130 was mucinous. In the case of the validation set, unselected for response to therapy, bivariate analysis with miRNA cluster expression and surgical debulking as covariates identified the chrXq27.3 miRNA cluster as a potential independent predictor of early relapse in type II EOC tumors (40/45 cases). This result was in silico validated on the TGCA miRNA profile [11] although the two platforms used for the analysis were different and a clear standardization in platform fabrication, assay protocol, and analysis methods is still lacking.

The finding that a lower expression of the miRNA cluster chrXq27.3 is consistently associated with shorter TTR also when the signature was challenged on a wider and independent publicly available dataset suggested a possible involvement of these miRNAs in cell growth and response to therapy. Members of this cluster have been previously identified as down-modulated in advanced-stage and/or high grade lesions [19], and one miRNA belonging to the cluster (miR-509) was found to be down-modulated in chemoresistant advanced-stage EOC [20], supporting the relevance of our results.

Our in vitro data essentially support the proposed antiproliferative role for these miRNA although a quite heterogeneous effect in terms of efficacy and efficiency has been observed when considered individually. In particular a role for miR-506 in favoring blockade of cell cycle in G1 phase and inducing cell death is suggested by our data. The role of miR-506 in tumor cells has not yet clearly defined and probably, as already described for other miRNAs, its effect could be cell and tumor type dependent. Indeed recent literature, in accordance with our observations, suggests a putative role as an anti-oncogenic miRNA in human bronchial epithelial cells where miR-506 expression regulates cell growth and proliferation [21] but also its over expression has been recently related to hydroxycamptothecin resistance in a colon cancer cell line by direct inhibiting expression of Peroxisome proliferator-activated receptors (PPARs) [22]. The other two miRNAs (miR-513a-5p and miR-513b) were selected among the other miRNAs belonging to the cluster on the basis of their differential fold change expression. However, their forced expression only partially reflects the effects observed with miR-506. Of note, two different analysis both performed on available unselected EOC samples (validation set and TCGA data) indicate that miR-513a-5p and miR-513b form a separate subcluster within the Xq.27.3 cluster. Taken together, expression and biological data suggest that within the identified cluster, the individual miRNAs could contribute to the overall cell behavior with only partially common mechanisms of regulation. The identification and validation of target genes of each individual members of the cluster could clarify this aspect and thus deserve further and specific investigation.

Eight out of 10 miRNAs, whose expression is down-regulated in patients with adverse outcome, localized in a less than 0.5Mb cluster on chrX. A variety of mechanisms including amplification, deletion or mutation may contribute to the deregulation of miRNA expression (reviewed in [23]). The primary regulatory mechanism of miRNA expression however, seems to be a transcriptional control essentially exerted by epigenetic silencing (reviewed in [24]). The availability of TGCA data prompted us to analyze in silico the methylation pattern of ChrX. Despite the continuous update of the platforms and the increase in the specific probes, still the coverage of our region of interest, i.e Xq27.3, is limited. However, we observed that the region Xq27- Xq28 was among the most frequently ipermethylated (Supplementary Figure 2). Although further analysis is needed, it seems likely that methylation play some role in the observed downregulation of miRNA cluster.

Due to the stringent criteria adopted for selection of training and test sets, we cannot exclude that, in addition to the validated miRNA cluster, other miRNAs are involved in drug response. Further studies using completely independent cohorts of patients from other institutions are clearly needed. In this context, the miRNA profiling of samples from a randomized clinical trial [25] is currently under investigation.

Taken together, the present data open the possibility of a new mechanistic hypothesis of gene regulation exerted by the chrXq27.3 miRNA cluster in EOC. The identified miRNA cluster, hopefully associated with other newly discovered miRNAs, may be applied in the context of clinical trials, as a potential marker of drug response in advanced-stage EOC patients.

## MATERIALS AND METHODS

### Patients and biological materials

A total of 130 patients with advanced-stage EOC were included in the study. All these patients underwent primary surgery at the Fondazione IRCCS Istituto Nazionale dei Tumori (INT) and had biological material available for molecular analysis, as well as complete clinical data and follow-up information. The Institutional Review Board approved the use of surgical specimens (immediately snap-frozen after surgery and stored at -80°C), formalin-fixed paraffin-embedded (FFPE) tissue blocks, and clinical data. The study was performed on tumor samples collected at surgery before chemotherapeutic treatment from three independent patient cohorts (training, test and validation sets) undergoing primary surgery between 1990 and 2008 and matched for time of diagnosis. All patients underwent exploratory laparotomy for diagnosis, staging, and debulking, and based on the extent of residual disease, patients were divided into three groups: no evident disease (NED); minimal residual disease (mRD, residual tumor <1 cm) and gross residual disease (GRD, residual tumor ≥1 cm). Patients NED or with mRD were considered optimally debulked (OD). Tumor staging was in accordance with the criteria of the International Federation of Gynecology and Obstetrics (FIGO). After surgery, patients received treatment with standard platinum-based therapeutic schedules (platinum without taxanes; platinum and paclitaxel) according to the time of accrual. Time to relapse (TTR) was the time in months from completion of chemotherapy until first evidence (clinical, instrumental or biological) of disease recurrence (if a complete response was achieved) or progression. Standard post-chemotherapy surveillance included routine physical examinations, serum CA-125 levels, and computed tomography scanning as clinically indicated. Follow-up time was the interval between diagnosis and date of death or the last information in medical records. Samples from training and validation sets were collected at the time of primary surgery and immediately frozen, samples from the test set were FFPE. Tumor content of all specimens was assessed by hematoxylin and eosin staining and all samples selected for miRNA analysis had >70% tumor cellularity and <20% necrosis. Patients from training (55 samples) and test (30 samples) sets were selected on the basis of residual disease after primary surgery [26]. To explore miRNA expression association with chemotherapy response, patients were also selected according to TTR as: patients with early relapse when the TTR was shorter than 12 (OD) or 6 (sub-OD) months; patients with late relapse when TTR was equal or longer than 36 (OD) or 12 (sub-OD) months. Patients in the validation set (45 samples) were selected from a consecutive case material on the basis of availability of both biological samples and clinical data without clinical restriction, but at an advanced-stage; patients reflected the age at diagnosis, stage, tumor grade, and surgical outcome of individuals typically diagnosed with advanced-stage EOC with a median TTR of 16 months [27]. Table 1 summarizes the clinical and pathologic characteristics of patients. Taking into account the new EOC classification [28], our case materials were mainly composed by Type II tumoros, indeed Type I tumors represented less than 10% in each set, being: 3 cases (1 low-grade serous and 2 clear cell tumors) present in training set; one low grade endometrioid tumor present in test set; and 4 cases (3 clear cell and 1 mucinous) in validation set. All these tumors were at stage III.

### miRNA analysis

RNA extraction from frozen or FFPE tissues, quality assessment, miRNA hybridization conditions, preprocessing, and miRNA data analysis were performed as detailed in the Supplementary material. Mature miRNAs were amplified [29] and labeled probes were hybridized on Illumina miRNA BeadChips Array. The microarray dataset and clinical information were deposited in the Gene Expression Omnibus database (experiment number GSE25204) according to MIAME (minimum information about a microarray experiment) guidelines. TaqMan microRNA assays from Applied Biosystems and Exiqon were used to quantify mature miRNAs. qRT-PCR validation and Taqman Assays are described in the Supplementary material.

### EOC cell lines and miRNA transfection

The OAW42 cell line (serous histotype, kindly provided by Dr. A. Ullrich, Max-Planck Institute, Germany, and verified in house for identity by microsatellite analysis) was maintained in MEM supplemented with 10% (v/v) fetal calf serum, 2 mmol/L L-glutamine (Sigma) and 1% non-essential amino acids (100X stock; Euroclone, Italy) in a humidified chamber (5% CO_2_, 37°C). The SKOV3 cell line, (serous histotype, from ATCC and verified in house for identity by microsatellite analysis) was maintained in RPMI 1640 (Sigma Aldrich) with 10% fetal calf serum (FCS) (Hyclone, Logan, UT) and 2 mmol/L glutamine in a 5% CO_2_ humidified atmosphere at 37°C. Cells were confirmed to be mycoplasma-free. Ectopic expression of miR-506, miR-513a-5p and miR-513b was pursued by exposing EOC cell lines to 20 nM miRNA precursors, purchased as a pre-miR molecule (Ambion, Austin,TX). Scrambled Pre-miR molecules (Pre-miR Negative Controls; Ambion) were used as a control. Transfection was carried out using Lipofectamine2000 (Invitrogen), according to the manufacturer’s protocol. The effect of ectopically expressed miRNAs was evaluated at day 3 after 4-h of transfection by assessing miRNAs levels by qRT-PCR.

### Proliferation assay and drug treatment

The day after miRNAs transfection, OAW42 cells were exposed for 7h to cis-platinum (DDP) (TEVA Italia s.r.l) at serial dilution starting from 10x10^−6^ to 0.03x10^−6^ M for Sulforhodamine B (SRB) assay and at 1x10^−6^ M, corresponding to inhibiting concentration 50 (IC_50_) according to our previous experience [30], for other drug treatment assays. The effects of miRNA transfection and DDP treatment on cell proliferation were assessed 72h after transfection by Trypan Blue exclusion assay or by SRB assay. For SRB assay cells were seeded in 96-well flat-bottom plates at an initial concentration of 5,000 cells per well in complete medium. Without removing the cell culture supernatant, cells were fixed by incubation for 1 h at 4°C with trichloroacetic acid (TCA) at the final concentration 10% (w/vol). Following extensive washing in water, cells were stained for 30 min with 0.4% (w/v) SRB dissolved in 1% acetic acid. Wells were rinsed with 1% acetic acid and air dried. Bound dye was solubilized with 10 mM Tris base (pH 10.5) in a shaker. The OD was measured at 550 nm in a microplate reader (BIORAD,model 550).

### Cell cycle evaluation

Cell cycle was analysed by flow cytometry. Cells were harvested in PBS containing 2 mM EDTA, washed once with PBS, fixed in iced ethanol 70% and incubated with 50mg/ml PI (Sigma) plus RNAse 0.5 mg/ml for 30 min at 4°C in the dark. Stained nuclei were analysed with a FACSCalibur (Becton & Dickinson), and the data analysed using ModFit cytometry Analysis Software (Becton-Dickinson).

### Bioinformatic analyses

Two publicly-available datasets reporting miRNA expression and clinical annotated data were identified at the best of our knowledge and downloaded from the web: GSE27290 (available on GEO repository, [31]) and the Cancer Genomics Atlas (TGCA, [11]) study available at http://tcga-data.nci.nih.gov/docs/publications/ov_2011/. The former data set consists of 62 diagnosed patients with stage III or IV serous ovarian cancer, while the latter reports the profiling of 489 high-grade serous EOCs. Although both studies were generated using the Agilent platform, GSE27290 was profiled on a pre-commercial version of miRNA chips (GPL7341) designed on miRBase 9.1. Due to the incomplete presence of miRNAs belonging to the chrXq27.3 cluster of our interest and the differences in miRBase annotation between GPL7341 and our Illumina platform, GSE27290 was not considered for further analyses. TGCA profiling was performed on 8x15Kv2 chip designed on miRBase10.1. Time to progression (defined as the interval from the date of initial surgical resection to the date of progression, date of recurrence, or date of last known contact if the patient was alive and has not recurred) was used as the primary end point.

### STATISTICAL ANALYSIS

Class comparison, hierarchical clustering, muldimensional scaling (MDS), global test of clustering, and heat map analyses were performed using BrB ArrayTools_v4.1.0-stable release (Simon, R. and Lam, AP; http://linus.nci.nih.gov/BRB-ArrayTools.html). Principal component analysis (PCA) was performed using the open-source PCA module of MeV (MultiExperiment Viewer, v4.6; http://mev.tm4.org), developed at The Institute for Genomic Research. The clinical and pathological characteristics of the three sets of patients were compared by Fisher’s exact test. Levels of miRNA and gene expression were compared by a Student’s t test. The linear relationship between variables was estimated by Pearson’s coefficient of correlation (r) and R^2^ coefficient of determination. We assessed the association of miRNA expression with TTR.

TTR curves were generated by the Kaplan Meyer method and differences between curves were compared using a non-parametric (log-rank) test and hazard ratios and 95% confidence intervals were also computed. A bivariable Cox regression model was used to evaluate the prognostic impact of miRNAs expression taking into account the effect of surgical debulking. A two level classification was used for both miRNAs expression and surgical debulking. *P* values of all statistical tests were two sided. GraphPadPrism v5 (GraphPad software, La Jolla US) and R statistical language version 2.11 (URL http://www.R-project.org) were used for statistical analyses.

## Supplementary Figures and Methods

Supplementary Figures

Supplementary Methods
