# Identification of a chrXq27.3 microRNA cluster associated with early relapse in advanced stage ovarian cancer patients

**DOI:** 10.18632/oncotarget.3998

**Published:** 2011-12-31

**Authors:** Marina Bagnoli, Loris De Cecco, Anna Granata, Roberta Nicoletti, Edoardo Marchesi, Paola Alberti, Barbara Valeri, Massimo Libra, Mattia Barbareschi, Francesco Raspagliesi, Delia Mezzanzanica, Silvana Canevari

Identification of a chrXq27.3 microRNA cluster associated with early relapse in advanced stage ovarian cancer patients

Marina Bagnoli, Loris De Cecco, Anna Granata, Roberta Nicoletti, Edoardo Marchesi, Paola Alberti, Barbara Valeri, Massimo Libra, Mattia Barbareschi, Francesco Raspagliesi, Delia Mezzanzanica and Silvana Canevari

Oncotarget 2011; 2: 1265 – 1278.

**Addendum**

We submitted to GEO repository new information to be added to the SuperSeries GSE25204 including: a new dataset, GSE67819, reporting the microRNA microarray profiling of our validation set; an update of the characteristic files including clinical data of patients in the GSE25202, GSE25203 and GSE67819 datasets.

