# Supplementary material for: Identification of a chrXq27.3 microRNA cluster associated with early relapse in advanced stage ovarian cancer patients
Source: Oncotarget. 2011 Dec 31;2(12):1265–78. doi: 10.18632/oncotarget.401 (PMC3282083; doi:10.18632/oncotarget.401)
Supplement: Supplementary Figures [file oncotarget-02-1265-s001.pdf]

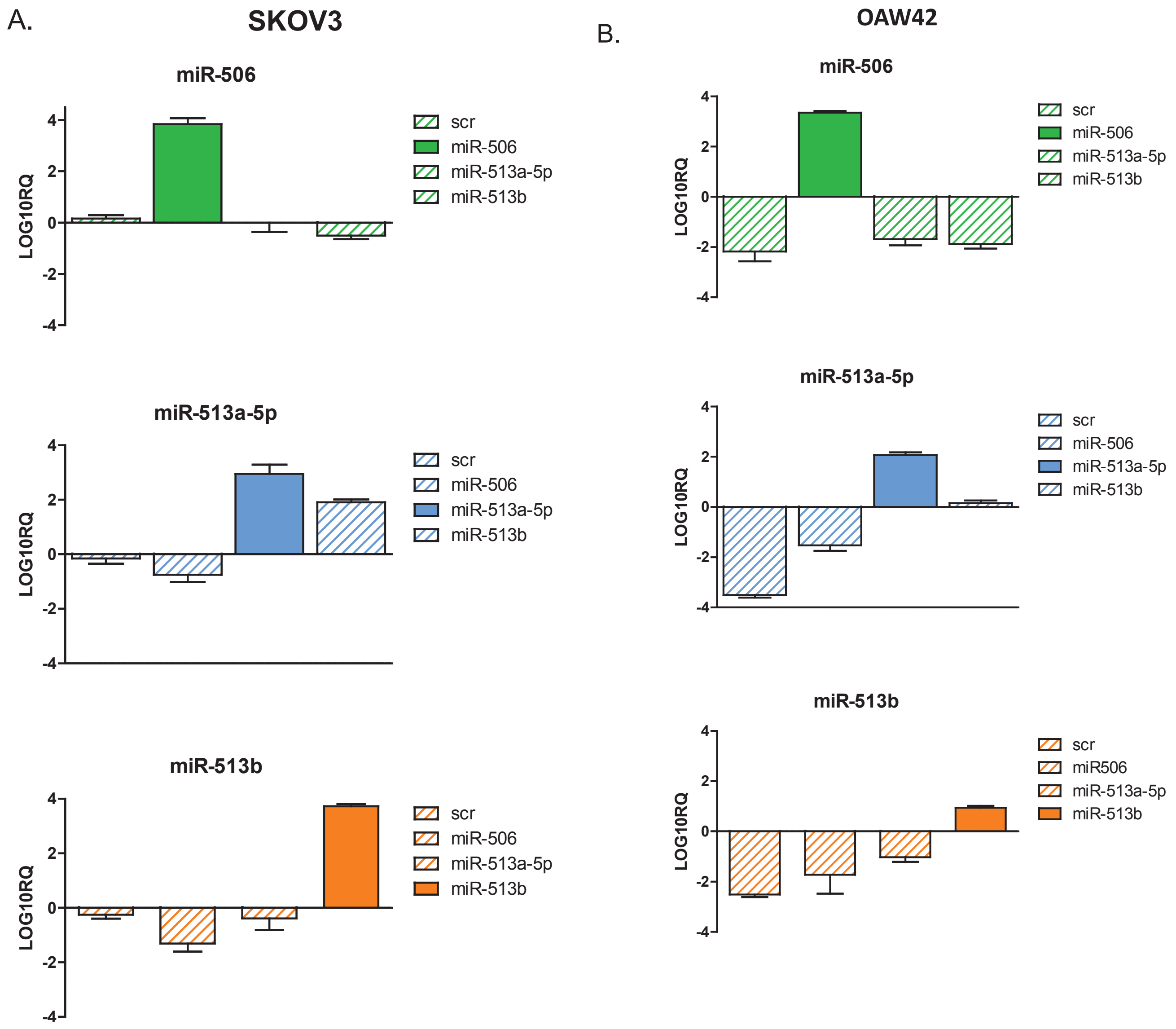

Supplementary Figure 1: ***Expression of transfected miRNAs by qRT-PCR.*** At 72h post transfection, specific miRNA expression was evaluated in SKOV3 (A) and OAW42 (B) cells by qRT-PCR using relative quantification (RQ) method (2exp(-delta(delta) Ct)). Value are expressed as LOG10 of the relative quantification value (LOG10RQ) and human total RNA was used as reference (LOG10RQ = 0). For each transfected miRNA, possible concurrent variations in expression of the other two miRNAs were considered. A partial cross-reactivity of miR-513a-5p probe with miR-513b was detected in both cell lines.

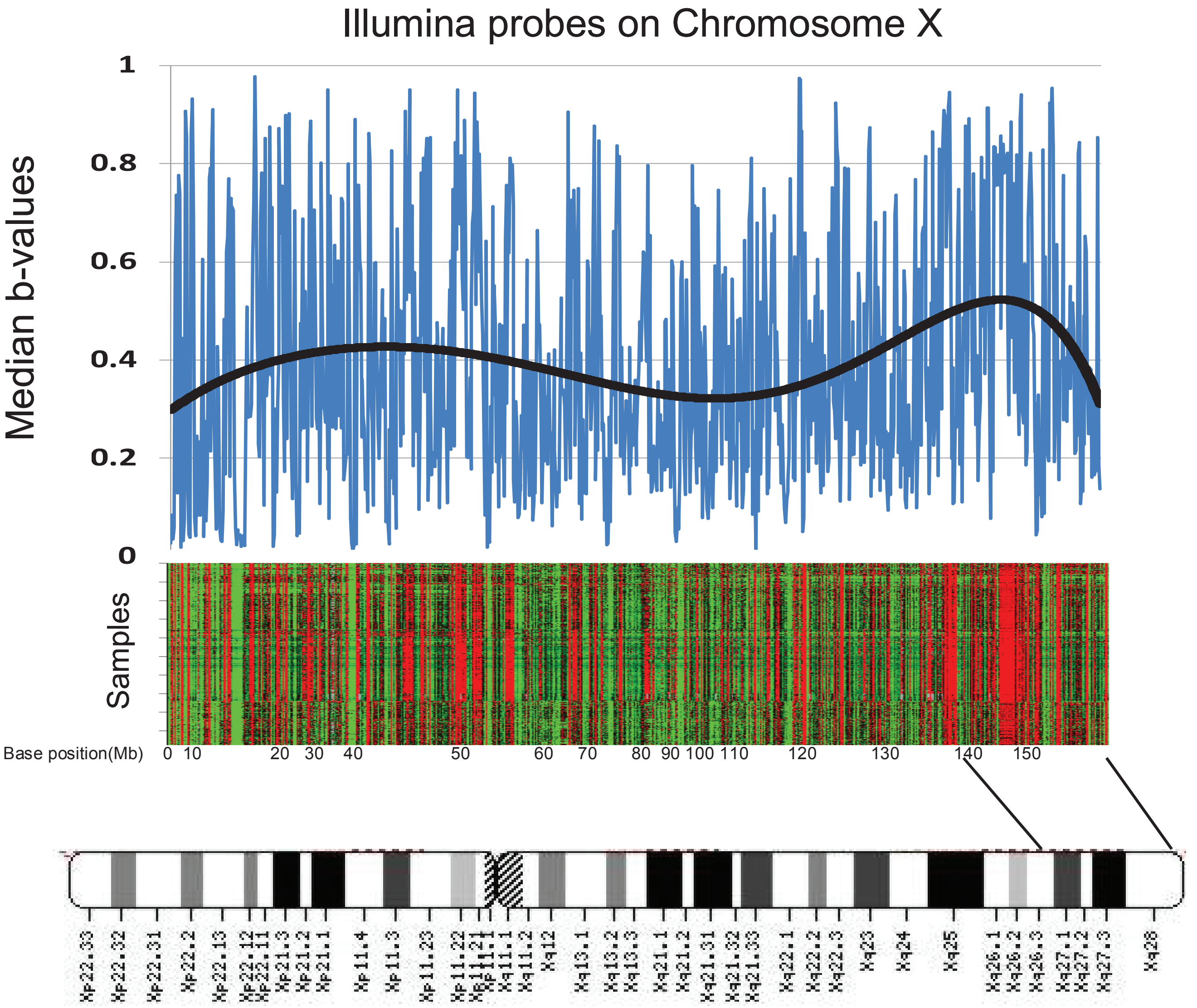

Supplementary Figure 2: ***Methylation of Chromosome X.*** Since epigenetic silencing has been reported as an important event in the inactivation of miRNA function in cancer, we investigated whether the chrX27.3 miRNA cluster expression might be under control of the methylation status and we take advantage of the methylation profile published by the Cancer Genome Atlas Research Network (see text reference 11) Methylation profiling data of 519 TGCA ovarian cancer samples was performed on Illumina Infinium HumanMethylation27 arrays covering 27,578 CpG sites across whole genome. Level 3 data containing the beta value calculations were downloaded from the TGCA Data Portal website (http://tgca.cancer.gov/dataportal). The beta value DNA methylation scores for each sample and locus were measured as M/(M+U) (M= intensity of the methylated, U= intensity of the unmethylated bead type for each CpG locus). The present analysis was restricted on stage III and IV samples used in our in silico miRNA validation and on the Illumina probes located on chrX (952 probes whose detection was significant imposing detection p-value>0.05) . The median beta value for each probe was plotted, sorted following their position along chrX and in order to appreciate the average trend inherent the data a polynomial regression curve was calculated. Only few probes cover chrXq27.3 region (cg05801573, cg12842316, cg03020597, cg27584469, cg07147350, cg08434396, cg12732953, and cg22495120). None of these probes were annotated as related to the transcription start site and functional promoter elements of our cluster of miRNAs. Although the coverage in the chrXq27.3 is limited, we observed that the flanking regions (Xq26.1, Xq26.2, Xq26.3, Xq27.1, Xq27.2, and Xq28) are the most frequent hypermethylated in chrX
