# Supplementary material for: Identification of a chrXq27.3 microRNA cluster associated with early relapse in advanced stage ovarian cancer patients
Source: Oncotarget. 2011 Dec 31;2(12):1265–78. doi: 10.18632/oncotarget.401 (PMC3282083; doi:10.18632/oncotarget.401)
Supplement: Supplementary Methods [file oncotarget-02-1265-s002.pdf]

Supplementary data: Identification of a chrXq27.3 microRNA cluster associated with early relapse in advanced stage ovarian cancer patients

miRNA isolation from frozen samples. For total RNA extraction, approximately 100 mg of frozen tissue was homogenized using a bench-top homogenizer (MM200, Retsch, Germany) in 1 mL of TRIzol Reagent (Invitrogen). Subsequent miRNA extraction was performed using the miRNeasy Mini Kit (Qiagen) according to the manufacturer's instructions. Qualitative analysis of RNA was performed using the Agilent 2100 Bioanalyzer, and 55/59 samples (93%) and 45/47 samples (97%) with good quality from the training and validation sets, respectively, were analyzed.

miRNA isolation from FFPE samples. Total RNA was extracted by processing four 20micron sections from FFPE tissue blocks using the RecoverAll Total Nucleic Acid Isolation kit (Ambion) according to the supplied protocol. Quantity and purity of RNA samples were assessed using a NanoDrop ND-100 spectrophotometer; 30/49 samples (61%) were considered adequate for miRNA profiling.

miRNA hybridization conditions. Mature miRNAs were amplified with the Illumina human_v2 MicroRNA expression profiling kit, based on the DASL (cDNA-mediated Annealing, Selection, Extension, and Ligation) assay, according to the manufacturer's instructions. Briefly, 800 ng/sample of total RNA was converted to cDNA and followed by annealing of a miRNA-specific oligonucleotide pool consisting of three parts: a universal PCR priming site at the 5' end, a sequence complementary to a capture sequence on the BeadArray and a miRNA-specific sequence at the 3' end. After PCR amplification and fluorescent labeling, probes were hybridized on Illumina miRNA BeadChips, allowing analysis of 1,146 sequences covering 97% of miRNAs described in the miRBase database v12.0. After hybridization, fluorescent signals were detected by the Illumina BeadArrayTM Reader.

Pre-processing and miRNA data analyses. Images were analyzed and primary data were collected with the supplied scanner software. Using BeadStudio software v3 and BeadStudio expression analysis module v3.3.8, the summary intensity given by the average bead intensities among all beads belonging to a particular miRNA was calculated along with the detection *P* value; data were quantile normalized and exported with neither background nor scale correction. Data matrices were filtered excluding signals with detection *P* values >0.05 and allowing 50% of missing values.

Quantitative reverse transcription-PCR (qRT-PCR) evaluation of miRNA expression. Primers were obtained from Applied Biosystems (Foster City, CA, USA) if available. Otherwise, assays were purchased from Exiqon (Vedbæk, Denmark).
TaqMan microRNA (Applied Biosystems) assays specific for hsa-miR-506 (Assay ID:1050), hsa-miR-507 (Assay ID:1051), hsa-miR-509-5p (Assay ID:2235), hsa-miR-513b (Assay ID:2757) and hsa-miR-335* (Assay ID: 2185) were used to detect and quantify mature miRNAs on an Applied Biosystems real-time PCR instrument in accordance with the manufacturer's instructions. Starting from 4-10 ng of total RNA, first strand cDNA was synthesized using miR-specific stem-loop primers and High-Capacity cDNA Reverse Transcription Kit (Applied Biosystems), reactions were run in a GeneAmp PCR 9700 thermocycler (Applied Biosystems) at

16°C for 30 minutes, 42°C for 30 minutes and 85°C for 5 minutes. The RT products, PCR master mix containing TaqMan 2× Universal PCR Master Mix (No Amperase UNG), and 10× TaqMan assay in 20 microL volume were amplified as follows: 95°C for 10 min followed by 40 cycles of 95°C for 15 sec and 60°C for 60 sec. Normalization was performed with the small nuclear RNA, RNU48 (Assay ID:1006).

qRT-PCR miRNA assays specific for hsa-miR-508-3p (Assay ID: 204480), hsa-miR-509-3p (204458), hsa-miR-513a-5p (Assay ID: 204785), and hsa-miR-514 (Assay ID: 204645) were purchased from Exiqon. qRT-PCR was performed using the miRCURY LNA™ Universal RT microRNA PCR system (Exiqon) and following the manufacturer's instructions. Twenty ng of total RNA was polyadenylated and reverse transcribed at 42°C (60 min) followed by heat-inactivation at 85°C (5 min) using a poly-T primer containing 5' universal tag. The resulting cDNA was diluted 80× and 8 microL used in 20 microL PCR amplification reactions as follows: 95°C for 10 min followed by 40 cycles of 95°C for 10 sec and 60°C for 60 sec.

Normalization was performed with SNORD48 (RNU48; Assay ID:203903). As a calibrator, FirstChoice Human Total RNA Survey Panel (Ambion) consisting of pools of total RNA from 20 different normal, human tissues (adipose, bladder, brain, cervix, colon, esophagus, heart, kidney, liver, lung, ovary, placenta, prostate, skeletal muscle, small intestine, spleen, testes, thymus, thyroid, trachea) was used. Each pool was comprised of RNA from at least 3 tissue donors. Total RNA was prepared from frozen or stored in RNAlater tissues, and after extraction purified RNA was treated with DNase.

miRNA expression levels were quantified using a sequence detection system (ABI Prism 7900HT; AppliedBiosystems) in triplicate and the threshold cycle (Ct) for each sample was determined. ABI SDS 2.4 software was used to analyze the data, and relative expression was calculated using the comparative DeltaCt method.
